# Resveratrol amplifies the anti-tumor effect of α-PD-1 by altering the intestinal microbiome and PGD2 content

**DOI:** 10.1080/19490976.2024.2447821

**Published:** 2024-12-30

**Authors:** Baohua Luo, Qingling An, Jingyu Lei, Dengxu Tan, Xiaoqiu Liu, Hui Li, Yong Zhao, Jing Qin, Caiqin Zhang, Yongbin Zhang, Changhong Shi

**Affiliations:** a Division of Cancer Biology, Laboratory Animal Center, Fourth Military Medical University, Xi’an, Shaanxi, P.R. China; b Department of Oncology, Guangdong Provincial Hospital of Chinese Medicine, Guangzhou, P.R. China; c School of Basic Medicine, Medical College of Yan’an University, Yan’an, P.R. China; d Science and Technology Innovation Center, Guangzhou University of Chinese Medicine, Guangzhou, P.R. China; e Laboratory Animal Center, Guangzhou University of Chinese Medicine, Guangzhou, P.R. China

**Keywords:** Resveratrol, immune checkpoint inhibitors, intestinal microbiota, prostaglandin D2, pancreatic cancer

## Abstract

Patients with pancreatic cancer (PC) receive limited benefits from immune checkpoint inhibitors (ICIs) alone. Recent studies have indicated that the combination of intestinal microflora regulators and ICI may be an effective strategy for enhancing the response to tumor immunotherapy. Resveratrol has been shown to regulate both intestinal microbiota and the immune microenvironment; therefore, we speculated that resveratrol may enhance the immunotherapeutic effect of α-PD-1 on PC. We constructed a double-humanized mouse model with both a humanized immune system and PC to evaluate the efficacy of different doses of resveratrol combined with anti-PD-1 mAb therapy and analyze the characteristics of the tumor immune microenvironment after treatment. In addition, I6S sequencing was performed on mouse fecal samples, and metabolomic analyses were performed on plasma samples to understand the characteristics of the intestinal microbiome and metabolites to explore the potential mechanism of anti-tumor immunity mediated by combined immunotherapy. Results suggested that the combination of resveratrol with anti-PD-1 mAb promoted the infiltration and activation of CD8^+^ T cells in tumor tissues, thus effectively inhibiting PC growth. Attentively, the effect emphasized the combined effect, dose-dependent nature and the involvement of gut microbiome. Furthermore, metabonomics showed that the combination therapy significantly increased the abundance of the metabolite prostaglandin D2 (PGD2), which was most likely related to the increased abundance of *Desulfovibrio fairfieldensis*. Finally, we confirmed that PGD2 amplified anti-tumor effect of α-PD-1 by activating prostaglandin D receptor 1 (DP1) to promote CD8 infiltration in double humanized PC mice and subcutaneously transplanted KPC mice. Overall, our data revealed resveratrol has the potential to enhance the efficacy of α-PD-1 by regulating the gut microbiome and its metabolites, and PGD2 was an effective molecular mediating the infiltration of CD8^+^T cells into tumor tissue to amplify the anti-tumor effect of α-PD-1.

**Significance:**

The anti-PD-1 mAb may be further considered along with PGD2 or active molecules that can promote PGD2 synthesis to enhance the anti-tumor immune response

## Introduction

Pancreatic cancer (PC) is a malignant tumor with rapid progression and poor prognosis, and its 5-year survival rate is only 8.5%.^1–3^ Most patients with PC are insensitive to radiotherapy, resistant to chemotherapy, and easily relapse after operation.^4–8^ Therefore, new therapeutic strategies for PC are urgently required to improve the therapeutic effect, prolong the survival time, and reduce the mortality rate. In the last decade, tumor immunotherapy, particularly immune checkpoint inhibitor (ICI) therapy, has revolutionized tumor treatment and brought new hope for prolonging the survival of patients with cancer. Unfortunately, patients with PC receive very limited benefits from ICIs alone.^9–14^ Therefore, an appropriate combined immunotherapy strategy may be a future direction for the clinical treatment of PC. In recent years, an increasing number of studies have found that the intestinal microbiota is an important factor affecting the anti-tumor efficacy of ICIs that can regulate the innate or acquired immune response in the tumor microenvironment in a variety of ways, thus affecting the anti-tumor efficacy of ICIs.^15–19^ Consequently, the combined application of intestinal microflora regulators and ICIs may be an effective new PC treatment method.

Studies have confirmed that resveratrol can regulate both the tumor immune microenvironment and the intestinal microbiome, indicating that it may amplify the effect of ICIs in the treatment of PC.^20–24^ However, how does resveratrol affect the tumor immune microenvironment, and whether the process is related to the intestinal microbiome has not been clarified. In this study, we constructed a humanized immune system model by transplanting screened frozen peripheral blood mononuclear cells (PBMCs) from healthy adults into severe combined immunodeficient mice after resuscitation and further injected human PC cells to obtain a double humanized mouse model. The anti-tumor efficacy of resveratrol combined with α-PD-1 therapy was evaluated, and the characteristics of the tumor immune microenvironment were analyzed after treatment in this mice model. In addition, we performed 16S sequencing of mouse fecal samples and metabolomics analyses of plasma samples to understand the characteristics of the intestinal microbiome and its metabolites to explore the potential mechanism of anti-tumor immunity mediated by the combined immunotherapy. We further validate our findings in the mouse PC transplantation model. These findings provide an ideal immunotherapy strategy for PC.

## Materials and methods

### Cells culture and clinical sample acquisition

The human PC cell line Bxpc3 (BCRC Cat# 60283, RRID:CVCL_0186) and mouse PC cell line Panc02 (NCI-DTP Cat# PAN 02, RRID:CVCL_D627) were purchased from the Cell Bank of the Chinese Academy of Sciences. The cells were cultured in RPMI-1640 (Gibco Cat# 11875093) medium supplemented with 10% fetal bovine serum (FBS, Gibco Cat# 10099141C) and 1% penicillin- streptomycin (Gibco Cat# 1037801) in a humidified incubator at 37°C in atmospheric air supplemented with 5% CO_2_. All human PBMCs were obtained from the Xijing Hospital of the Fourth Military Medical University (FMMU) and were approved by the Institutional Review Board (IRB) of FMMU (KY20203128–1).

### Animal experiment

Female NOD-*Prkdc*^*em26Cd52*^*Il2rg*^*em26Cd22*^/Gpt (NCG) mice, C57BL/6J, *LSL-Kras*^*G12D/+*^*LSL-Trp53*^*R172H/+*^ (KP) mice and *pdx1-Cre* mice were purchased from GemPharmatech LLC (China). Spontaneous pancreatic cancer mouse model (KPC) was obtained after mating with KP mice and *pdx1-Cre* mice. The pancreatic tumor tissue was used to establish transplantation model. All these mice models were housed in a specific pathogen free environment at the Laboratory Animal Center of FMMU and randomly assigned for the experiment. All animal experimental protocols were approved by the Institutional Animal Care and Use Committee of FMMU (No. 20200602).

### Screening of PBMC donors

Fresh PBMCs from different healthy volunteers were cryopreserved for subsequent experimental selection. Frozen PBMCs (2 × 10^7^/µL) from different donors were injected into the tail vein of NCG mice after resuscitation for the reconstruction of human immune system. A cryopreservation solution (Procell, Cat# PB180436) was employed for standard cryopreservation and recovery procedures. 1 week after PBMCs implantation, the human PC cell line Bxpc3 (2 × 10^6^/200 µL) were subcutaneously implanted into mice. 4 weeks after PBMC implantation, we evaluated the reconstitution of human immune system and tumor growth. We believed a donor with a higher level of reconstruction and less inhibitory effect on tumor growth would be more appropriate. We would select the donor for subsequent efficacy evaluation experiments.

### Construction of the double humanized pancreatic cancer mouse model and administration strategy

Different doses of resveratrol (100 mg/kg and 200 mg/kg, TCI Cat# R0071) were administered by gavage starting 2 weeks before subcutaneously implanting the human PC cell line Bxpc3 (2 × 10^6^/200 µL) in NCG mice until the end of the experiment. The screened frozen PBMCs (2 × 10^7^/µL) were injected into the tail vein of mice after resuscitation for the reconstruction of human immune system 1 week before tumor cell implantation. Three weeks after PBMC implantation, peripheral blood was collected from the mice to assess the reconstitution of the human immune system by analyzing the proportion of human CD45^+^ cells using flow cytometry. Generally, the proportion of human CD45^+^ cells in all immune cells of mouse peripheral blood was more than 25%, indicating successful construction of the human immune system.^25,26^ After the human immune system was successfully reconstructed in NCG tumor-bearing mice, and the tumor reached a volume of approximately 100 mm^3^, α-PD-1 (pembrolizumab, BioXCell Cat# SIM0010, RRID:AB_2894731) therapy was started by intraperitoneal injection (200 µg/per mouse) and performed twice a week for three consecutive weeks. Tumor growth was dynamically monitored after the tumor cells were implanted, and tumor volume was monitored twice per week. Tumor volume was calculated as:V (mm^3^) = length (mm) × width^2^ (mm^2^)/2.

### Antibiotic treatment

Two weeks before the implantation of PC cells, as we began to administer 200 mg/kg resveratrol intragastrically, a mixture of antibiotics consisting of 1 g/L neomycin (Macklin Cat# N814740), 0.5 g/L vancomycin (Macklin Cat# V820413) and 1 g/L ampicillin (Macklin Cat# A800429) was added to the daily water provided to the mice at the same time until the end of the experiment.^27–30^

### Construction of the subcutaneously transplanted KPC mouse model and administration strategy

After KP mice and *pdx1-Cre* mice were fed adaptively for one week respectively, they were caged together for natural mating. *Pdx1*^*Cre*^*LSL-Kras*^*G12D/+*^*LSL-Trp53*^*R172H/+*^ (KPC) mice were selected from their pups by genotype identification. When KPC mice developed orthotopic pancreatic cancer, the orthotopic tumor tissue was obtained and cut into 1 mm^3^ tissues, which were then subcutaneously transplanted into C57BL/6J mice to construct subcutaneously transplanted KPC mouse model. Treatment was started the next day. Prostaglandin D2 (PGD2, MCE Cat# HY-101988) was intraperitoneally injected once a day (1 mg/kg) and α-PD-1 (BioXCell Cat# BE0146, RRID: AB_10949053) was administered twice a week (10 mg/kg) until the end of the experiment. Tumor volume was dynamically monitored after the tumor tissues were implanted. The experiment ended when the maximum tumor volume reached 1000 mm^3^.

### Flow cytometry

At 3 weeks after PBMC implantation, 100 µL of peripheral blood from mice with reconstituted immune systems was collected by the tail amputation method into an anticoagulant centrifuge tube, to which 1 mL of phosphate-buffered saline (PBS, Gibco Cat# 20012027) was previously added. PBMCs were isolated and purified using the Ficoll density gradient centrifugation method, and a single-cell suspension was prepared. The sample volume was fixed to 100 µL. Next, anti-human FITC-CD45 (BD Biosciences Cat# 555482, RRID: AB_395874) and anti-mouse PECY5-CD45 antibodies (BD Biosciences Cat# 561870, RRID: AB_10898351) were incubated according to the flow cytometry instructions. After incubation, 1 mL of PBS was added to dilute the antibodies. After centrifugation, the supernatant was discarded, and then the cells were suspended with 500 µL of PBS buffer and placed on ice. The reconstruction level of human immune cells in mouse peripheral blood was evaluated using FlowJo10.0 software (RRID:SCR_008520) and the hCD45/(hCD45 + mCD45) calculation method.

### Histology

Tumor and colon tissues were dissected and partially embedded in 4% paraformaldehyde for 48 h. Paraffin-embedded tissue sections (5 µm) were stained with H&E. The slides were scanned and observed under a Leica optical microscope.

### Immunofluorescence

The resected tumor tissues were fixed in 4% paraformaldehyde and embedded in paraffin. The sections were deparaffinized, rehydrated, and boiled in a microwave for 20 min in 10 mm citrate buffer for antigen retrieval. IHC was performed using anti-human CD45 (Abcam Cat# ab40763, RRID:AB_726545, dilution rate:1:200), CD8 (Proteintech Cat# 66868–1-Ig, RRID:AB_2882205, dilution rate:1:16000), GZMB (Abcam Cat# ab208586, RRID:AB_ 2,924,920, dilution rate:1:500), CD4 (Abcam Cat# ab133616, RRID:AB_2750883, dilution rate:1:500), PD-L1 (Abcam Cat# ab205921, RRID:AB_2687878, dilution rate:1:500), Ki67 (Abcam Cat# ab16667, RRID:AB_ 302,459, dilution rate:1:200) and PECAM-1 (Proteintech Cat# 80530–1-RR, RRID: AB_2918900, dilution rate:1:500). Sections were incubated overnight at 4°C and then with the appropriate Alexa Fluor-conjugated secondary antibodies. Slides were washed between staining steps with Bond Wash and stripped between each round of staining by heat treatment in antigen retrieval buffer. Nuclei were counterstained with DAPI. Data were acquired by sequential acquisition, and tile-scan imaging was performed using a Leica SP8 LIGHTNING confocal microscope (RRID:SCR_018169).

### Culture of intestinal microbiota

At the end of the treatment period, the feces of the mice in the different treatment groups were collected. Samples (0.2 g) from each mouse were dissolved in 1 mL of sterile saline and diluted 10,000 times after mixing. Further, 10 µL of the solution was collected and dropped into the center of a blood agar plate, and colonies were calculated after culturing in a 37°C incubator for 24 h.

### PCR analyses of Desulfovibrio

Fresh dry feces of mice in different treatment groups were dissolved in 1 ml sterile distilled water, crushed and homogenized to form suspension, and then amplified by PCR. The primers were 16SRNA-F: ATGGCTGTCGTCAGCT, 16SRNA-R: ACGGGCGGTGTGTAC, *Desulfovibrio-*F: CCGTAGATATCTGGAGGAACATCAG, *Desulfovibrio-*R: ACATCTAGCATCCATCGTTTACACAGC.

#### Desulfovibrio fairfieldensis gavage strategy

After the double humanized pancreatic cancer mouse model was successfully constructed, *Desulfovibrio fairfieldensis* (1×10^9^ CFU/100 µL, Sweden, Uddevalla Cat# B285169) was intragaxed to every mouse every other day until the end of the experiment. Peripheral blood plasma of mice in different treatment groups was collected to detect the abundance of PGD2 according to the PGD2 ELISA Kit (Elabscience Cat# E-EL-0066) procedure.

### Arachidonic acid analysis

Fresh dry feces of mice in different treatment groups were dissolved in 1 ml anhydrous ethanol after weighing, and then crushed and homogenized into suspension. The suspension was centrifuged and the supernatant was subjected to high performance liquid chromatography (HPLC). Chromatographic method: Instrument: Hitachi chromaster analytical high performance liquid chromatograph; Column: Huapu XAqua C18 (4.6 × 250 mm, 5 µm, 100A); Mobile phase A: ultrapure water (0.1% trifluoroacetic acid), mobile phase B: acetonitrile (0.1% trifluoroacetic acid); Chromatographic conditions: 80% of the phase B at a constant flow rate of 1 mL/min, the detection wavelength is 215 nm, the injection volume was 20 µL.

### 16S rRNA sequencing

Microbial DNA was isolated from the feces of the mice using a Qiagen MagAttract power microbiome kit. The V4 region of the 16S rRNA-encoding gene was amplified from the extracted DNA using barcoded dual-index primers. And then the PCR assay was conducted. The size of the amplicon library (approximately 399 bp) was confirmed using an Agilent 2100 Bioanalyzer Instrument (RRID:SCR_018043). The pooled amplicon library was sequenced on an Illumina MiSeq platform using a 500-cycle MiSeq V2 Reagent Kit according to the manufacturer’s instructions, with modifications to the primer set. The “Preparing Libraries for Sequencing on the MiSeq” (Illumina MiSeq System, RRID:SCR_016379) protocol was used to prepare libraries and obtained FASTQ files. Raw microbial 16S rRNA gene sequencing data were analyzed using Mothur (version 1.40.5; running the 64 bit version; RRID:SCR_011947). Silva reference files (release 132) were used to align the sequences, and the open-reference operational taxonomic unit picking protocol was used with 97% sequence identity. The data processing steps based on the MiSeq standard operating procedure.^31^ The pcr.seqs command aligned the sequences to the reference alignment (silva. nr_v132. align). To classify sequences, a Bayesian classifier with the classify.seqs command was used with a cutoff value of 80, and silva.nr_v132.align and silva.nr_v132.tax were used as the reference and taxonomy, respectively.

### Metabolomic profiling

Mouse blood samples were centrifuged at 3000 rpm for 15 min, and the supernatants were collected. The samples were derivatized and analyzed using ultra-performance liquid chromatography-mass spectrometry (UPLC-MS, RRID:SCR_022052). The metabolomics data analysis was conducted with RStudio (RRID:SCR_000432) and online versions of MetaboAnalyst (RRID:SCR_015539, http://www.metaboanalyst.ca). Partial least square discriminant analysis (PLS-DA) and principal component analysis (PCA) were performed using the R package mixOmics (RRID:SCR_016889). *p* values in both PCA and PLS-DA plot were calculated by permutational multivariate analysis of variance (PERMANOVA) using distance matrices through the R package vegan. Differential metabolites analysis were conducted using the R package MetaboAnalystR (RRID:SCR_016723). The significantly altered metabolites were determined by variable importance in projection (VIP) scores from pairwise PLS-DA analysis and pairwise comparisons using the Wilcoxon rank-sum test. Benjamini-Hochberg false-discovery rate (FDR) was used to correct for multiple comparison. Metabolites with VIP score > 1 and *p* values < 0.05 were considered significant. Interactions were estimated by Spearman’s rank correlation. Metabolite set enrichment analysis (MSEA) was performed using the online tool MetaboAnalyst.

### Biogenic analysis

Tumor Immune Estimation Resource (TIMER, RRID:SCR_018737, http://cistrome. org/TIMER/, accessed on 28 July 2021) was used to determine the relationship between PTGDS expression and immune infiltration. The TIMER database contains 10,897 samples across 32 cancer types from The Cancer Genome Atlas (TCGA, RRID:SCR_003193) database to allow for the evaluation of immune infiltration abundance. The CIBERSORT algorithm (RRID:SCR_016955) was used to calculate the relative proportion of immune cell infiltration in the TCGA-PAAD RNA-sequencing samples, and Pearson’s test was performed to examine the correlation between PTGDS expression and the relative proportion of multiple immune cells. Pearson’s *p* values < 0.5 were considered statistically significant.

### Statistical analysis

All data were expressed as the mean ± SEM. Differences between groups were analyzed using one-way ANOVA analysis of variance, followed multiple comparisons post-hoc test for the least significant differences. The data were approximately normally distributed, and the variance was similar between the groups. The critical *p* value for significant differences was set to 0.05. Statistical analyses were performed using GraphPad Prism V .8.0.2 (RRID:SCR_002798). ^ns^*p* > 0.05, *p < 0.05, **p < 0.01, ***p < 0.001.

## Results

### Potent anti-tumor efficacy of 200 mg/kg resveratrol plus α-PD-1 therapy

The human leukocyte antigen (HLA) matching between tumor cells and transplanted immune cells was an essential prerequisite for eliciting tumor antigen-specific T cell responses. Therefore, before constructing double humanized PC mouse model, we first identified whether the HLA of PBMC from different donors matched with the pancreatic cancer cell line Bxpc3. We reconstituted mice by resuscitating frozen PBMCs from different donors to reconstructe human immune system, further transplanted tumor cells, and then evaluated the level of reconstitution and tumor growth so as to identified the histocompatibility between tumor cells and PBMCs (Fig S1). We believed a donor with higher level of reconstruction and less inhibitory effect on tumor growth would be more appropriate, which indicated that there was better HLA matching and less rejection between PBMCs and tumor cells. Among all donors, donor 3 and donor 4 displayed higher level of reconstruction and less inhibitory effect on tumor growth. Furthermore, the tumor size of these two groups of mice was similar to that of the mice in the non-reconstructed group (Fig S1), indicating that PBMC had little effect on tumor growth and could reflect the effect of drug therapy to the greatest extent. So we selected the two donors to constructed double humanized PC mouse models and administered different drug interventions to evaluate the anti-tumor efficacy of the different treatment strategies. Attentively, the toxicity and reactivity of drugs at different doses are of concern, especially at high doses. Therefore, we then gave NCG mice continuous gavage of different doses of resveratrol for 6 weeks to determine the potential toxicity threshold by analyzing the mucosal inflammation of colon tissue. The results showed that continuous administration of 300 mg/kg resveratrol induced lymphocyte proliferation in colon tissue. It means that this dose may cause colon inflammatory damage, which suggested a degree of enterotoxicity. While, no significant abnormalities were observed in colon tissue after continuous intragastric administration of 100 mg/kg and 200 mg/kg resveratrol. So, we selected the doses of 100 mg/kg and 200 mg/kg for follow-up experiments.

**Figure 1.**
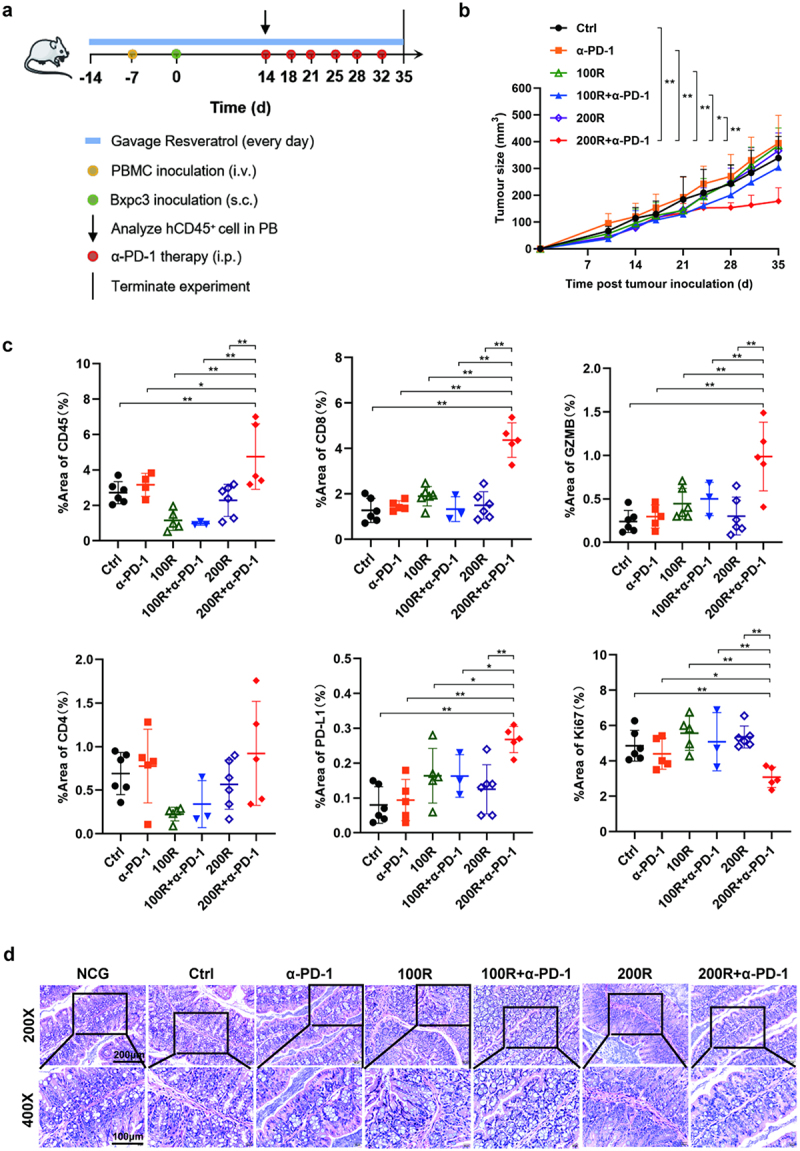
Potent antitumor efficacy of 200 mg/kg resveratrol plus α-PD-1 therapy. (a) Strategy for the construction of double humanized pancreatic cancer mouse model and administration. (b) Tumor growth curves of mice in different treatment groups. (c) Quantitative analysis of the positive area per field by ImageJ software (NIH) using ImmunoRatio plugin. (d) Representative images of the structural characteristics of colonic tissues in different treatment groups at 200× and 400× magnification.

The detailed time nodes for the different processes are shown in the figure (Figure 1a).^22,32–34^ The reconstitution of the human immune system by analyzing the proportion of human CD45^+^ cells using flow cytometry (Fig. S3). The results showed that 200 mg/kg resveratrol plus α-PD-1 therapy significantly slowed PC growth. Interestingly, 100 mg/kg resveratrol plus α-PD-1 therapy exhibited only a modest anti-tumor effect and was not effective at slowing tumor growth. In parallel, 200 mg/kg resveratrol, 100 mg/kg resveratrol, and α-PD-1 monotherapy presented a similar effect and barely inhibited PC growth (Figure 1b). These results emphasized the importance of combination therapy, and whether the combination therapy of resveratrol and α-PD-1 could mediate the anti-tumor effect was closely related to the dose of resveratrol. Furthermore, we analyzed the immunological changes in the tumor tissues by immunofluorescence staining (Figure 1c, S4). We observed that compared to the other groups, the content of CD45^+^ cells, CD8^+^ T cells and functional cytokine GZMB among CD8^+^ T cells in the 200 mg/kg resveratrol plus α-PD-1 group distinctly increased. However, significant differences in the content of CD4^+^ T cells were not observed among all groups. The function of CD8^+^ T cells may be the primary factor that leads to the anti-tumor immune response mediated by 200 mg/kg resveratrol plus α-PD-1 therapy, while the role of CD4^+^ T cells in this process is not so significant. Studies have indicated that the efficacy of ICIs targeting PD-1/PD-L1 immune checkpoints may be related to the expression levels of PD-L1 in tumor tissues. When PD-L1 is highly expressed, patients with cancer may be more responsive to anti-PD-1 mAb treatment.^35^ Therefore, we also tested the expression level of PD-L1 in tumor tissues after different treatments and found that 200 mg/kg resveratrol plus α-PD-1 therapy distinctly increased the content of PD-L1, which may be associated with amplified anti-tumor efficacy. Finally, we analyzed the expression of Ki67, a marker of cell proliferation, and found that the 200 mg/kg resveratrol plus α-PD-1 therapy reduced the expression of Ki67 in tumor tissue, thus effectively slowing tumor proliferation. In brief, resveratrol combined with α-PD-1 therapy was able to regulate the tumor immune microenvironment. It may boost the anti-tumor immune response by promoting the infiltration and activation of CD8^+^ T cells and expression of PD-L1 in tumor tissues, thus effectively inhibiting PC growth. In addition, we also observed the mucosal structure of the mouse colon after different treatment strategies. We found that there was no structural destruction or lymphocyte proliferation of the colonic mucosa in all groups (Figure 1d). As a consequence, we suggest that the dose of 200 mg/kg resveratrol has the potential to enhance the anti-tumor efficacy of α-PD-1and without significant toxicity.

### Mice in different treatments had varying compositions of gut microbiome

**Figure 2.**
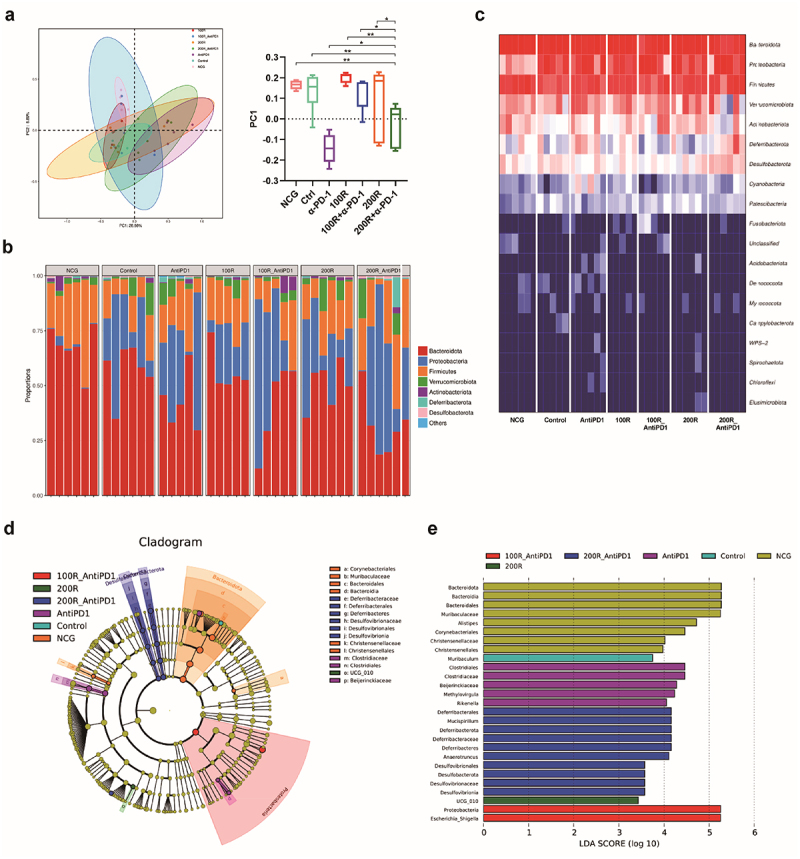
Mice in different treatments had varying compositions of gut microbiome. (a) Principal coordinate analysis (PCoA) showed that different treatment strategies induced different clustering of the microbial community structure. (b) Bar chart showed that different treatment groups of mice had different intestinal microbiota composition. (c) Heat map showed that different treatment groups of mice had different intestinal microbiota composition. (d, e) difference analysis showing that different treatment groups of mice had different dominant flora.

**Figure 3.**
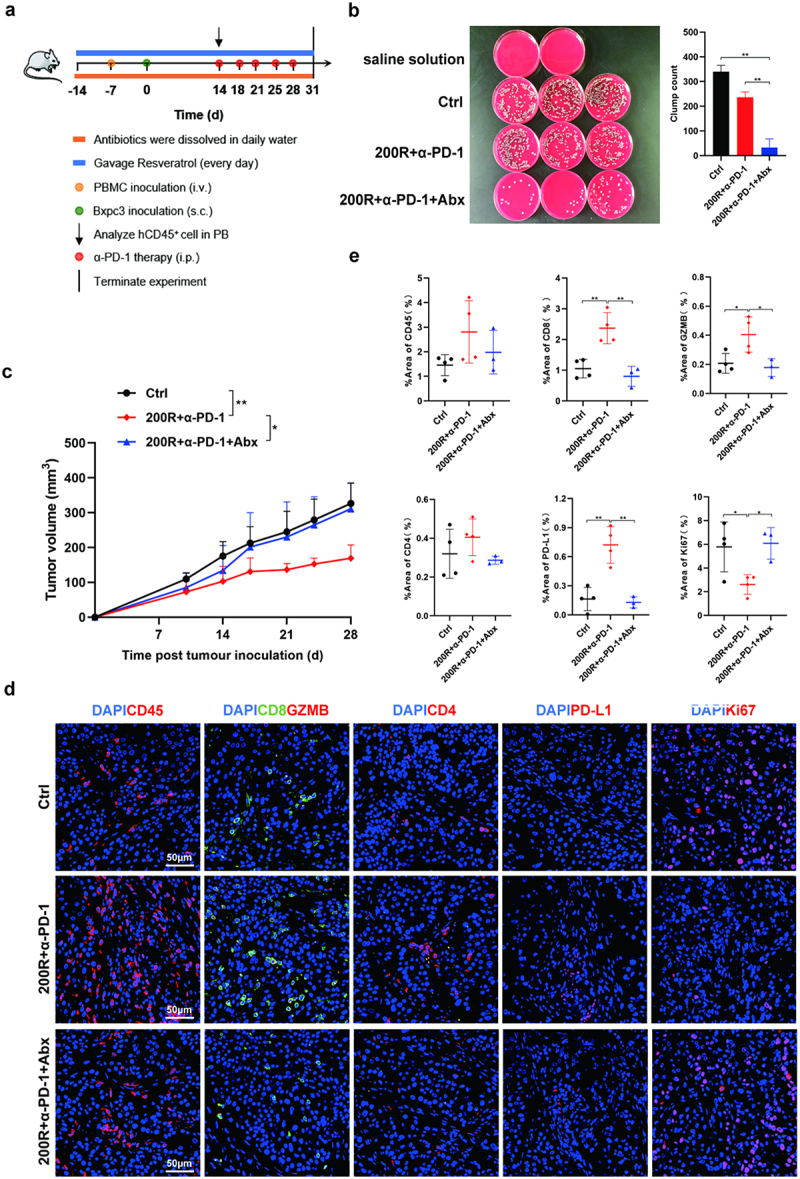
Mixed antibiotics attenuated the antitumor effect of 200 mg/kg resveratrol plus α-PD-1 therapy. (a) Strategies for the construction of double humanized pancreatic cancer mouse model and administration. (b) Characteristics of colony abundance in different treatment groups and corresponding quantitative results. (c) Tumor growth curves of mice in different treatment groups. (d) Representative images of tumor immune microenvironment characteristics in different treatment groups of mice at 200× magnification. (e) Quantitative analysis of positive area per field by ImageJ software (NIH) using the ImmunoRatio plugin.

As mentioned above, resveratrol not only has the potential to regulate the tumor immune microenvironment but also the intestinal microbiota.^20–24^ Therefore, we further analyzed the characteristics of the intestinal microbiota before and after the different treatments. We individually collected fecal samples from the mice before administering all interventions for this experiment and two weeks after the different treatments. Next, 16S ribosomal RNA gene sequencing was performed on fecal samples. The results demonstrated that different treatment strategies induced different clustering of the microbial community structure, which represented the β-diversity, as shown by the principal coordinate analysis methods (Figure 2a). These findings suggest that the differently treated mice had different gut microbiota compositions (Figure 3b,c). Further analysis revealed that different treatment groups had specific dominant microbiomes (Figure 3d,e). Consequently, we infer that the effective tumor inhibition and enhanced tumor immune response mediated by 200 mg/kg resveratrol plus α-PD-1 therapy might be associated with alterations in the intestinal microbiome.

### Role of resveratrol in promoting the anti-tumor immune effects of α-PD-1 was dependent on gut microbiome involvement

To further clarify whether the effective tumor inhibition and enhanced tumor immune response mediated by 200 mg/kg resveratrol plus α-PD-1 therapy were associated with intestinal microbiota or whether other mechanisms besides the intestinal microbiome were involved, we treated mice with 200 mg/kg resveratrol plus α-PD-1 and added a mixture of antibiotics to the daily water to eliminate the intestinal microflora in mice. Subsequently, we observed whether the efficacy of 200 mg/kg resveratrol plus α-PD-1 therapy in the treatment of PC was affected by the combined antibiotic therapy (Figure 3a).^27^ The results indicated that the oral antibiotic mixtures effectively reduced the richness of the intestinal microbiota (Figure 3b). With the sharp decrease of the abundance of intestinal microflora, the original inhibitory effect of 200 mg/kg resveratrol plus α-PD-1 therapy on PC growth was offset (Figure 3c). An analysis of the tumor immune microenvironment showed that antibiotics obviously reduced the ability of 200 mg/kg resveratrol plus α-PD-1 therapy to promote CD8^+^ T cell infiltration and activation and PD-L1 expression in tumor tissue; moreover, antibiotics enhanced tumor cell proliferation (Figure 3d,e). Based on the above results, we believe that the effective tumor inhibition and enhanced tumor immune response mediated by 200 mg/kg resveratrol plus α-PD-1 therapy were dependent on gut microbiome involvement.

### Amplified tumor immune response mediated by 200 mg/kg resveratrol plus α-PD-1 therapy may be due to metabolite prostaglandin D2 (PGD2)

**Figure 4.**
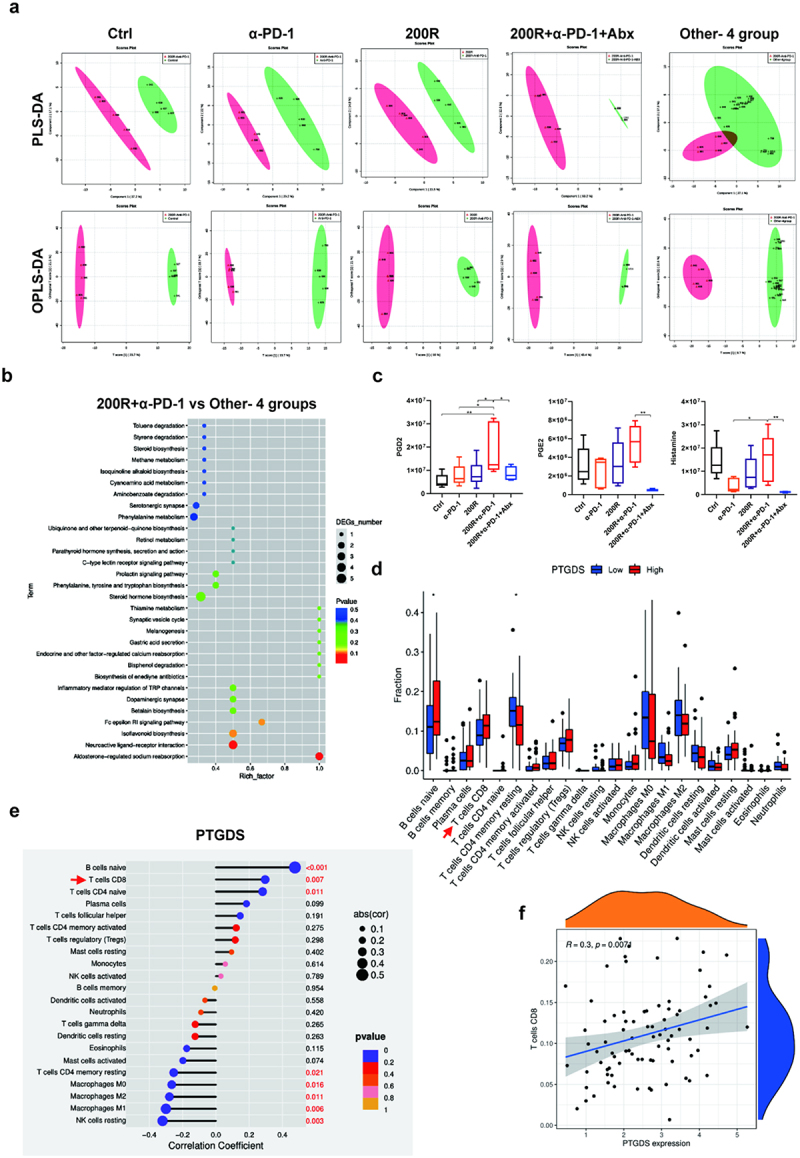
Amplified tumor immune response mediated by 200 mg/kg resveratrol plus α-PD-1 therapy may be due to metabolite PGD2. (a) Partial least squares discriminant analysis (PLS-DA) and orthogonal partial least squares discriminant analysis (OPLS-DA) showed that after 200 mg/kg resveratrol plus α-PD-1 therapy, specific clustering of the microbial metabolite structure formed that was significantly different from that of the other treatment strategies. (b) Pathway analysis of differential metabolites between the 200 mg/kg resveratrol plus α-PD-1 therapy group and other groups. (c) Contents of PGD2, PGE2, and histamine in serum of mice after different treatments. (d) High- and low-expression groups were divided according to the median expression levels of PTGDS genes, and relative proportions of different immune cells between the two groups were compared. (e) Correlations between PTGDS expression and immune cells infiltration using tumor immune. (f) Correlations between PTGDS expression and relative proportions of CD8^+^ T cells.

To further clarify how the gut microbiome influences tumor immunotherapy, we performed a metabolomic analysis on plasma samples from different treatment groups to understand the characteristics of intestinal microbiome metabolites and explore the potential underlying mechanisms.^28–30,36^ We first analyzed whether there were differences in the metabolites of the intestinal microbiota after the different treatments. According to partial least squares discriminant analysis (PLS-DA) and orthogonal partial least squares discriminant analysis (OPLS-DA), specific clustering of the microbial metabolite structure was observed after 200 mg/kg resveratrol plus α-PD-1 therapy, and this clustering was significantly different from that after other treatment strategies (Figure 4a). Next, we compared the abundance levels of corresponding metabolites in the different treatment groups and screened out the differential metabolites in the 200 mg/kg resveratrol plus α-PD-1 therapy group, which were either increased or decreased compared with that in the other treatment groups. We then performed a KEGG analysis of these differential metabolites to identify the associated metabolic pathways. For further screening of the pathways, we performed a comprehensive analysis of the pathways involved in the differential metabolites, including enrichment and topological analyses, and identified the key pathways showing the highest correlation with the differential metabolites. Among them, two pathways may be closely related to immunology: the Fc epsilon RI signaling pathway and the inflammatory mediator regulation of TRP channels, which involve the main differential metabolites PGD2, prostaglandin E2, and histamine (Figure 4b,c). PGD2 was the most abundant among the differentially expressed metabolites. Compared with the other treatment strategies, the content of PGD2 in the serum of mice increased significantly after 200 mg/kg resveratrol plus α-PD-1 therapy (Figure 4c). Based on the above results, we speculated that 200 mg/kg resveratrol plus α-PD-1 therapy amplified the anti-tumor immune response by altering the intestinal microbiome composition and upregulating the metabolite PGD2 in serum.

To further clarify the underlying mechanisms, we focused on the possible association between PGD2 in pancreatic tumor tissues and the tumor immune microenvironment. We performed a biogenic analysis and found that the content of prostaglandin D2 synthase (PTGDS) was correlated with the abundance of some immune cells in tumor tissues (Figure 4d,e). Interestingly, it was positively correlated with the abundance of CD8^+^ T cells in the pancreatic tumor tissues (Figure 4e,f), suggesting that PTGDS may promote the infiltration of CD8^+^ T cells into the pancreatic tumor tissue. Interestingly, PTGDS is a key enzyme that catalyzes the synthesis of PGD2. Above all, it was reasonable to speculate that PGD2 may be an effective molecular mediating the infiltration of CD8^+^T cells into PC tissue to amplify the anti-tumor effect of α-PD-1.

### PGD2 amplified anti-tumor effect of α-PD-1 in double humanized PC mice and subcutaneously transplanted KPC mice

**Figure 5.**
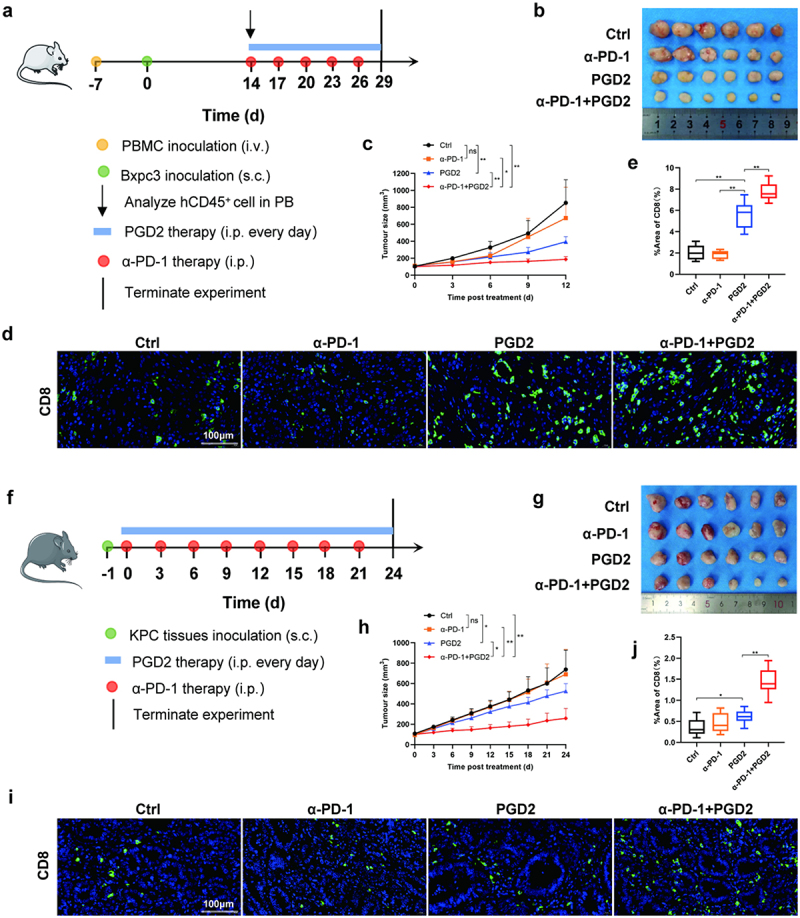
PGD2 amplified anti-tumor effect of α-PD-1 in multiple mouse models. (a) Strategy for the construction of double humanized PC mouse model and administration. (b) Tumor tissues of mice in different treatment groups. (c) Tumor growth curves of mice in different treatment groups. (d) Representative images of CD8^+^ T cell in different treatment groups of mice at 400× magnification. (e) Quantitative analysis of the positive area per field by ImageJ software (NIH) using ImmunoRatio plugin. (f) Strategy for the construction of subcutaneously transplanted KPC mouse model and administration. (g) Tumor tissues of mice in different treatment groups. (h) Tumor growth curves of mice in different treatment groups. (i) Representative images of CD8^+^ T cell in different treatment groups of mice at 400× magnification. (j) Quantitative analysis of the positive area per field by ImageJ software (NIH) using ImmunoRatio plugin.

To test above hypothesis, we injected PGD2 and α-PD-1 intraperitoneally into double humanized PC mice and evaluated their antitumor effects (Figure 5a). The results showed that, consistent with previous results, α-PD-1 was not effective in inhibiting the growth of PC. However, PGD2 combined with α-PD-1 effectively inhibited tumor growth and the effect was more obvious than that of PGD2 alone (Figure 5b,c). Then, we analyzed the abundance of CD8^+^T cells in PC tissues of different treatment groups and found that it was the highest in the combined treatment group (Figure 5d,e). Considering that the absence of some immune cells in tumor immune microenvironment of double humanized PC mice may affect the experimental results, we used subcutaneously transplanted KPC mice with perfect immune system for further verification and got consistent results (Figure 5f–j). The mice with Panc02 tumor also showed similar results (Fig. S5). Above all, it was reasonable to think that PGD2 amplified the anti-tumor effect of α-PD-1 by promoting the infiltration of CD8^+^T into tumor tissues.

### Desulfovibrio fairfieldensis increased the abundance of PGD2 and amplified anti-tumor effect of α-PD-1

**Figure 6.**
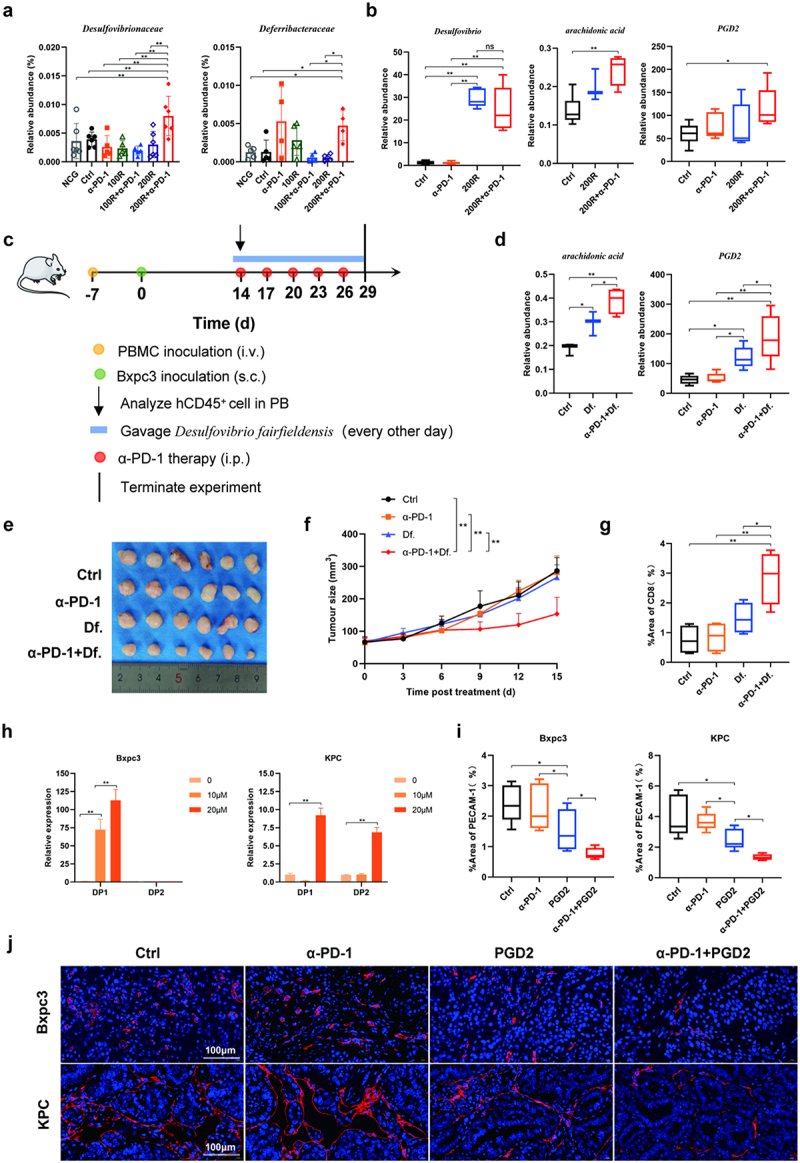
*Desulfovibrio fairfieldensis* increased the abundance of PGD2 and amplified anti-tumor effect of α-PD-1. (a) Quantitative analysis of dominant flora in 200 mg/kg resveratrol plus α–PD-1 therapy group. (b) Quantitative analysis of the abundance of *Desulfovibrio*, arachidonic acid and PGD2. (c) Strategy for the construction of double humanized PC mouse model and administration. (d) Quantitative analysis of the abundance of arachidonic acid and PGD2. (e) Tumor tissues of mice in different treatment groups. (f) Tumor growth curves of mice in different treatment groups. (g) Quantitative analysis of the positive area per field by ImageJ software (NIH) using ImmunoRatio plugin. (h) The expression of DP in Bxpc3 cells and KPC cells after different doses of PGD2 stimulated. (i) Quantitative analysis of the positive area per field by ImageJ software (NIH) using ImmunoRatio plugin. (j) Representative images of PECAM-1^+^ cell in different treatment groups of double humanized PC mice and KPC mice at 400× magnification.

Based on the above, we speculated that 200 mg/kg resveratrol plus α-PD-1 therapy may promote the infiltration of CD8^+^ T cells in tumor tissues by altering the intestinal microbiome composition and upregulating PGD2, which amplifies the anti-tumor effect. However, which gut microbiota mediated the increase of PGD2 and how PGD2 promoted the infiltration of CD8^+^T into tumor tissues remains to be further clarified. Based on 16s sequencing, difference analysis showed that compared with the other treatment groups, the abundance of *Desulfovibrionaceae* and *Deferribacteraceae* in the 200 mg/kg resveratrol plus α-PD-1 therapy group was significantly increased, with *Desulfovibrionaceae* being more prominent and represented a dominant bacterial family (Figure 6a). So, we hypothesized that the 200 mg/kg resveratrol plus α-PD-1 therapy increase the level of PGD2 may be related to the increased abundance of a genus under the *Desulfovibrionaceae*. PGD2 was metabolized by arachidonic acid (AA).^37^ It has been reported that there was a significant positive correlation between *desulfovibrio* and AA metabolism.^38,39^ So, *desulfovibrio* may increased abundance of PGD2. We conducted additional PCR-based analyses on the bacterial composition in the stool samples of mice following resveratrol treatment. Our findings revealed a significant increase in the abundance of the *Desulfovibrio* genus post-treatment. This enhancement was notably more pronounced when resveratrol was administered in conjunction with α-PD-1. Consistently, the levels of AA in feces and PGD2 in peripheral blood plasma were significantly elevated following treatment with 200 mg/kg of resveratrol combined with α-PD-1. While there was a noticeable trend toward increased levels of AA and PGD2 with resveratrol monotherapy, the increase was not substantial enough to achieve statistical significance. Consequently, resveratrol alone treatment was insufficient to enhance the infiltration of CD8^+^ T cells into tumor tissues by significantly elevating PGD2 levels (Figure 6b). Consequently, we believed that this combination treatment strategy increased the abundance of *desulfovibrio*, which promoted AA metabolism and thus increased PGD2 synthesis. According to previous literature reports, three species of *desulfovibrio* have been isolated from human specimens: *D. desulfuricans, D. fairfieldensis*, and *D. piger*, of which *D. fairfieldensis* was reported to be able to promote AA metabolism.^40–42^ To verify this, we gavage *Desulfovibrio fairfieldensis* to double humanized PC mice and measure the abundance of AA and PGD2 (Figure 6c,d). The results showed that *Desulfovibrio fairfieldensis* could indeed increase the abundance of AA and promote the synthesis of PGD2, and this effect was more significant when combined with α-PD-1, which could promote the infiltration of CD8^+^T into tumor tissues and effectively inhibit tumor growth (Fig. d-g, S6). As a consequence, we believe that 200 mg/kg resveratrol plus α-PD-1 therapy increased the abundance of *Desulfovibrio fairfieldensis* which promoted AA metabolism and up-regulated the abundance of PGD2, while PGD2 promoted the antitumor effects of α-PD-1 by promoting the infiltration of CD8^+^T into tumor tissues.

Next, we explored how PGD2 promoted the infiltration of CD8^+^T into tumor tissues. PGD2 has two authentic receptors, D prostanoid (DP1) and chemoattractant receptor homologous molecule expressed on Th2 cells (DP2).^43^ We stimulated Bxpc3 cells and KPC cells with different doses of PGD2 in vitro, and found that PGD2 could promote DP1 expression in both two cells, but could not promote DP2 expression in Bxpc3 cells (Figure 6h). Therefore, we believe that PGD2 may mediate the infiltration of CD8^+^T into tumor tissues mainly through DP1. Studies have shown that PGD2 can inhibit angiogenesis in tumor tissues and reduce the release of immunosuppressive factors by activating DP1, thus slowing down tumor growth.^44,45^ We detected platelet endothelial cell adhesion molecule-1 (PECAM-1) in tumor tissues of double humanized PC mice and subcutaneously transplanted KPC mice after PGD2 treatment, and found that PGD2 could reduce the expression of pecam-1, and the effect was more significant after combining with α-PD-1. Therefore, we hypothesized that PGD2 inhibits angiogenesis by activating DP1, thereby reversing the tumor immunosuppressive microenvironment and enhancing the infiltration of CD8^+^T into tumor tissues.

## Discussion

In the past decade, although immunotherapy, especially ICIs targeting PD-1/PD-L1, has shown encouraging efficacy in some clinical tumor protocols,^46–51^ its response rate in the treatment of PC has been extremely low. Combination therapy is an immunotherapy developed for PC. In this study, we constructed a double humanized PC mouse mode by implanting allogeneic human PBMC and PC cell lines to evaluate the anti-tumor efficacy of combination therapy in PC. Attentively, the HLA matching between allogeneic tumor cells and immune cells is an essential prerequisite for eliciting tumor antigen-specific T cell responses.^52^ So, we first identified the histocompatibility between tumor cells and PBMCs. A donor of PBMCs with higher level of reconstruction and less inhibitory effect on tumor growth may be more appropriate, which indicated that there was better HLA matching and less rejection between immune cells and tumor cells. We selected these donors to constructed double humanized PC mice for related experiments to minimize the impact of HLA mismatch on immunotherapy. However, it is undeniable that utilization of identical patient-derived immune cells and tumor xenografts is a more ideal approach.

CD8^+^ T cells are the key cells that play an anti-tumor role in the body. In this study, we observed that 200 mg/kg resveratrol plus α-PD-1 therapy could significantly promote the infiltration and activation of CD8^+^ T cells but had no significant effect on CD4^+^ T cells, indicating that the enhanced tumor immune response mediated by the combination therapy was mainly related to the function of CD8^+^ T cells, which was consistent with the previous research results.^53^ In addition, 200 mg/kg resveratrol plus α-PD-1 therapy also increased the expression of PD-L1 in tumor tissue. PD-L1 is a negative immune regulatory molecule that promotes the escape of tumor cells from immune surveillance. High expression of PD-L1 in tumor tissues is often thought to suppress the immune response and promote tumor growth. However, in the context of ICIs therapy, research has shown that the efficacy of ICIs targeting the PD-1/PD-L1 axis may correlate with the expression levels of PD-L1 within tumor tissues. Specifically, when PD-L1 expression is elevated, cancer patients tend to exhibit a heightened response to anti-PD-1 monoclonal antibody therapy.^35^ Furthermore, certain studies have suggested that the expression of PD-L1 in tumor tissues is upregulated as a result of antitumor immune responses. When tumor-infiltrating lymphocytes recognize tumor antigens and initiate anti-tumor immune responses, tumor cells counter by upregulating the expression of PD-L1. This increased PD-L1 expression allows tumor cells to bind to PD-1 on T cells, thereby inducing adaptive immune resistance and evading the cytotoxic effects of immune cells. It is evident that the surface expression of PD-L1 on tumor cells is intricately linked to the T cell-mediated anti-tumor immune response; hence, the elevated expression of PD-L1 can also be considered as a biomarker indicative of tumor immune activation.^54,55^ Ki67 is a nuclear antigen that is associated with cells in the process of proliferation, and its expression level serves as an indicator of the degree of cellular proliferation activity. A higher positive rate of Ki67 is indicative of more rapid tumor growth.

Previous studies have confirmed that resveratrol can regulate not only the tumor immune microenvironment but also the intestinal microflora.^20–24^ However, the interactions between these factors remain unclear. It is also uncertain whether mediated regulation of the tumor immune microenvironment is related to the intestinal microflora. In this study, through the use of antibiotics to eliminate intestinal microflora in mice, it was found that the role of resveratrol in promoting the anti-tumor immune effects of α-PD-1 was dependent on the involvement of the gut microbiome and was most likely related to the increased abundance of *Desulfovibrio fairfieldensis*. Microbiome-derived metabolites may be key messengers between the gut microbiota and host immune system.^56^ To explore the potential mechanism between the intestinal microbiota and the tumor immune microenvironment, we conducted a metabonomic analysis of mouse serum samples after different treatments to identify the possible molecular targets. The results showed that PGD2 content was significantly higher in the 200 mg/kg resveratrol plus α-PD-1 group than in the other treatment groups. By biosignal analysis,^57^ we found that PGD2 may be an effective molecular mediator of CD8^+^ T cell infiltration into tumor tissue to amplify the anti-tumor effect of α-PD-1. And then, we confirmed our suspicions by intraperitoneal injection of PGD2 into double humanized PC mice and mouse PC transplantation model (KPC) with complete immune system.

However, the specific molecular mechanism underlying the interaction between PGD2 and CD8^+^ T cells remains unclear. PGD2 is a small lipid molecule with two receptors, prostaglandin D2 receptor (DP) and Th2 cell chemotactic receptor homologue (CRTH2), exerting its effects through ligand-receptor binding.^37^ In our study, we discovered that PGD2 can suppress the expression of PECAM-1 by activating DP1, which in turn inhibits angiogenesis and facilitates the infiltration of CD8+ T cells into tumor tissues. However, a more comprehensive exploration of the underlying mechanisms remains to be conducted. A previous study showed that Interleukin-33 (IL33) activated Group 2 innate lymphoid cells (ILC2s) in tumor tissue by binding to its receptor ST2 on ILC2 to produce CCL5, potentially recruit CD103^+^ DCs into tumors, and activated CD8^+^ T cells to amplify the efficacy of anti-PD-1 immunotherapy.^58^ Interestingly, ILC2 also expresses CRTH2, the receptor of PGD2. PGD2 may activate ILC2s in tumor tissue by binding to the receptor CRTH2 to initiate a mechanism similar to that in the above study, thus promoting the infiltration of CD8^+^ T cells into tumor tissue and enhancing the efficacy of α-PD-1. In summary, there are many possibilities for the potential mechanism of PGD2 promoting tumor immune response that need to be further explored.In our study, It is clear that PGD2 does promote the infiltration of CD8+T into tumor tissues to amplify the anti-tumor effect of α-PD-1.

## Conclusions

**Figure 7.**
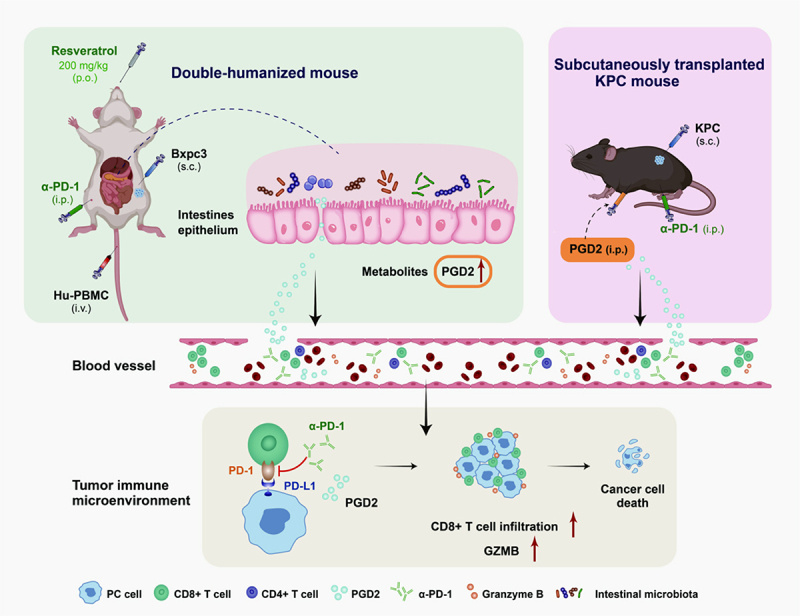
PGD2 promotes the infiltration of CD8^+^ T cells into tumor tissues to amplify anti-tumor effect of α-PD-1.

In summary, this study confirmed that resveratrol combined with anti-PD-1mAb has the potential to enhance anti-tumor immune responses and effectively slow PC growth by Increasing the abundance of *Desulfovibrio fairfieldensis*. In addition, PGD2 may be an effective molecular mediator of CD8^+^ T cell infiltration into tumor tissues to amplify anti-tumor effect of α-PD-1 (Figure 7). Consequently, the anti-PD-1mAb should be further considered along with the addition of PGD2 or active molecules that can promote PGD2 synthesis to enhance the anti-tumor response.

## Supplementary Material

Supplemental Material

## Data Availability

The data within this study are available from the corresponding author on reasonable request.

## List of abbreviations

PBS: phosphate-buffered saline
PC: pancreatic cancer
ICI: immune checkpoint inhibitor
PGD2: prostaglandin D2
DP1: prostaglandin D receptor 1
PBMCs: peripheral blood mononuclear cells
FMMU: Fourth Military Medical University
IHC: immunohistochemical
PLS-DA: partial least squares discriminant analysis
OPLS-DA: orthogonal partial least squares discriminant analysis
PTGDS: prostaglandin D2 synthase
HLA: human leukocyte antigen
AA: arachidonic acid
PECAM-1: platelet endothelial cell adhesion molecule-1.
