# Supplementary material for: Resveratrol amplifies the anti-tumor effect of α-PD-1 by altering the intestinal microbiome and PGD2 content
Source: Gut Microbes. 2024 Dec 30;17(1):2447821. doi: 10.1080/19490976.2024.2447821 (PMC12931716; doi:10.1080/19490976.2024.2447821)
Supplement: Supplemental Material [file KGMI_A_2447821_SM3318.zip › Revised Supplementary.docx]

**Supplementary**

**Time (d)**

**0**

**7**

**14**

**21**

**28**

PBMC inoculation (i.v.)

Bxpc3 inoculation (s.c.)

Analyze hCD45^+^ cell in PB


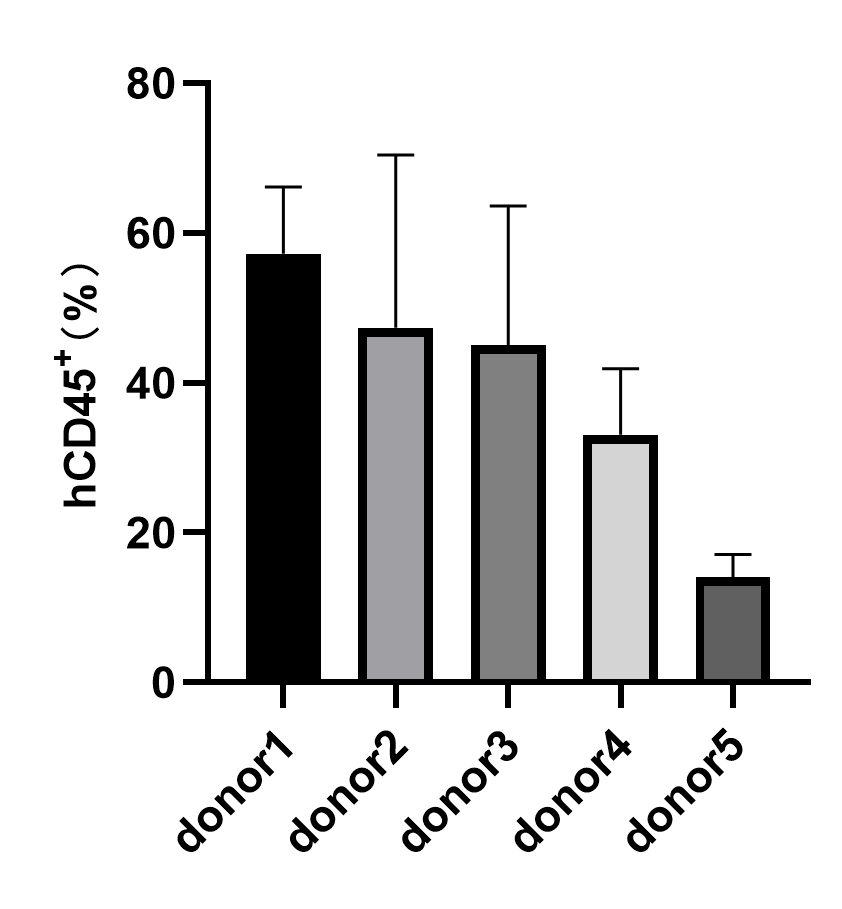

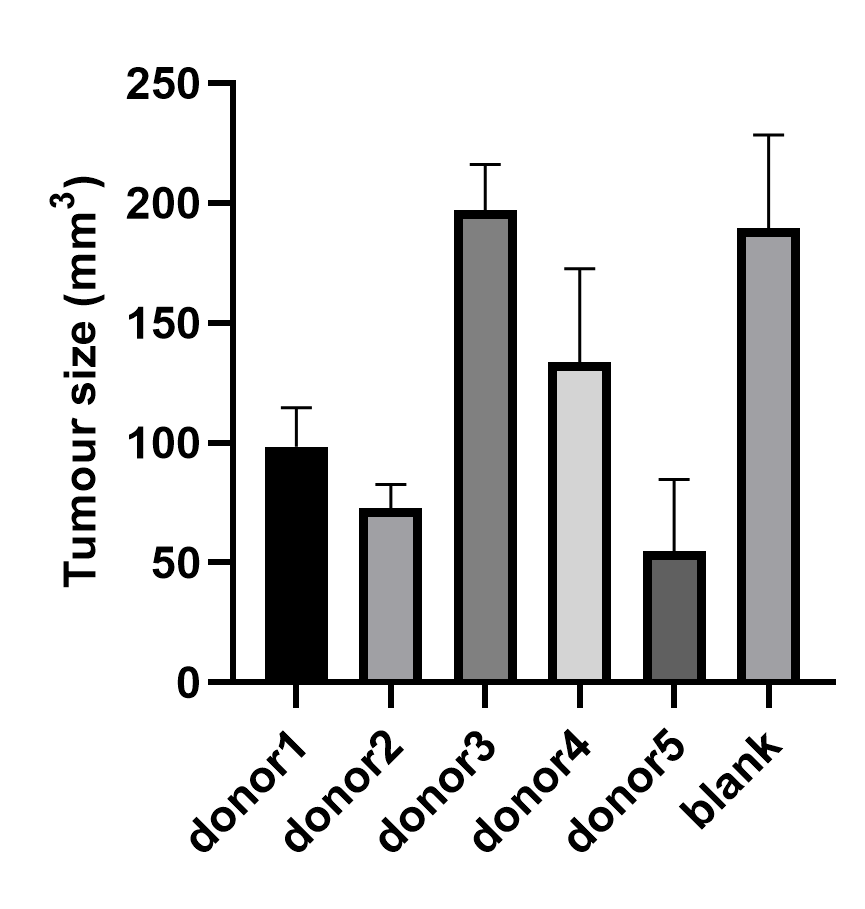


**Supplementary Fig. S1 Identified whether the HLA of PBMC from different donors matched with the pancreatic cancer cell line Bxpc3.** Reconstituted mice by resuscitating frozen PBMCs from different donors to reconstructe human immune system, further transplanted tumor cells, and then evaluated the level of reconstitution and tumor growth so as to identified the histocompatibility between tumor cells and PBMCs.


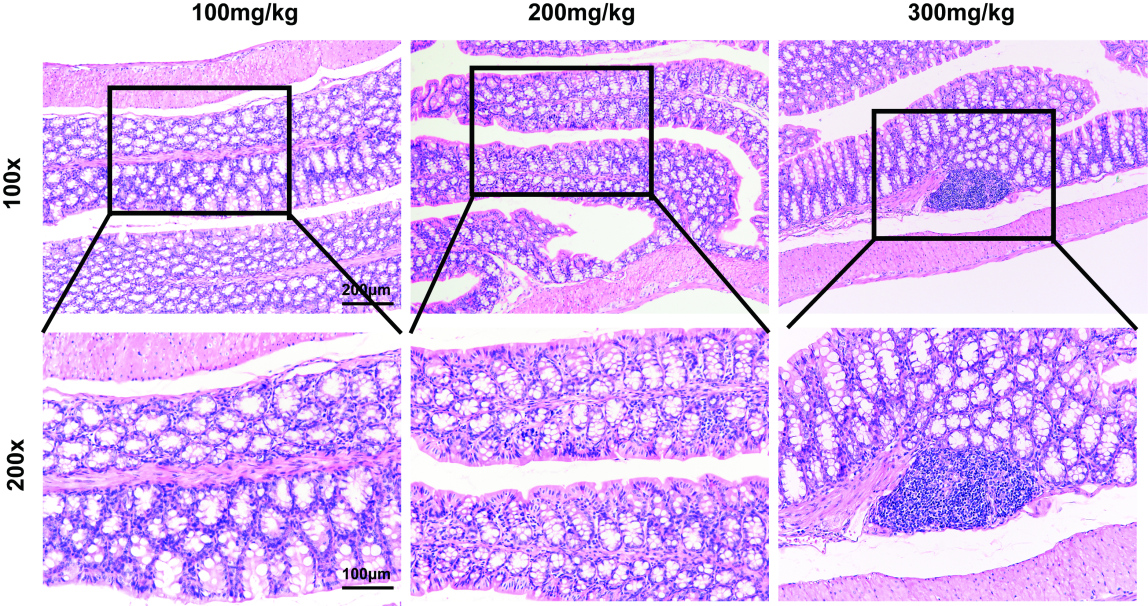


**Supplementary Fig. S2 Representative images of the structural characteristics of colonic tissues after different doses of resveratrol treatment at 100× and 200× magnification.**

**Supplementary Fig. S3 loop-gate strategy for flow cytometry.** From left to right, circles indicate all PBMCs of mice, human CD45^+^ cells and mouse CD45^+^ cells from the PBMCs, and human CD45^+^ cells from all CD45^+^ cells.

**
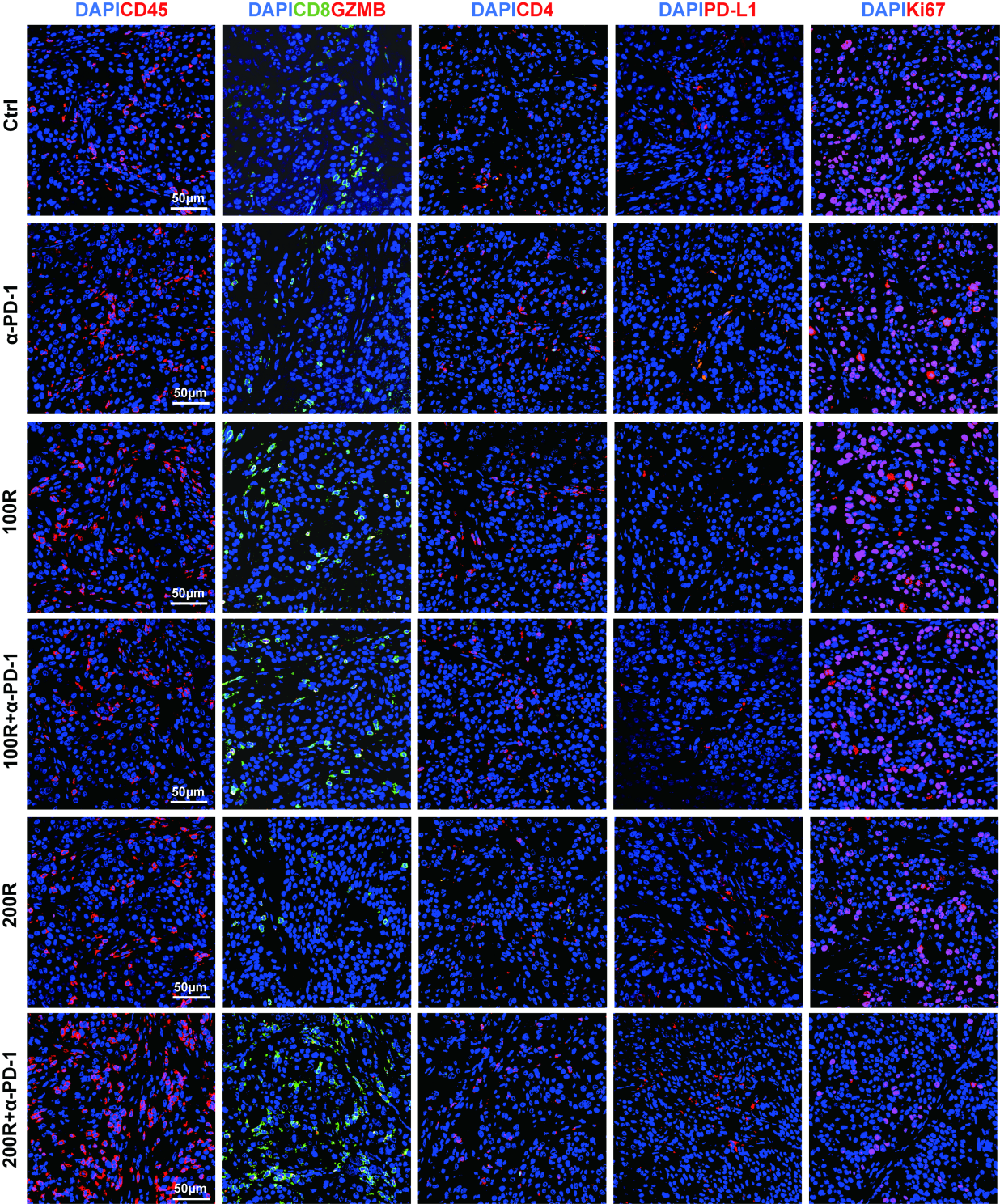
**

**Supplementary Fig. S4 Representative images of tumor immune microenvironment characteristics in different treatment groups of mice at 200× magnification.**


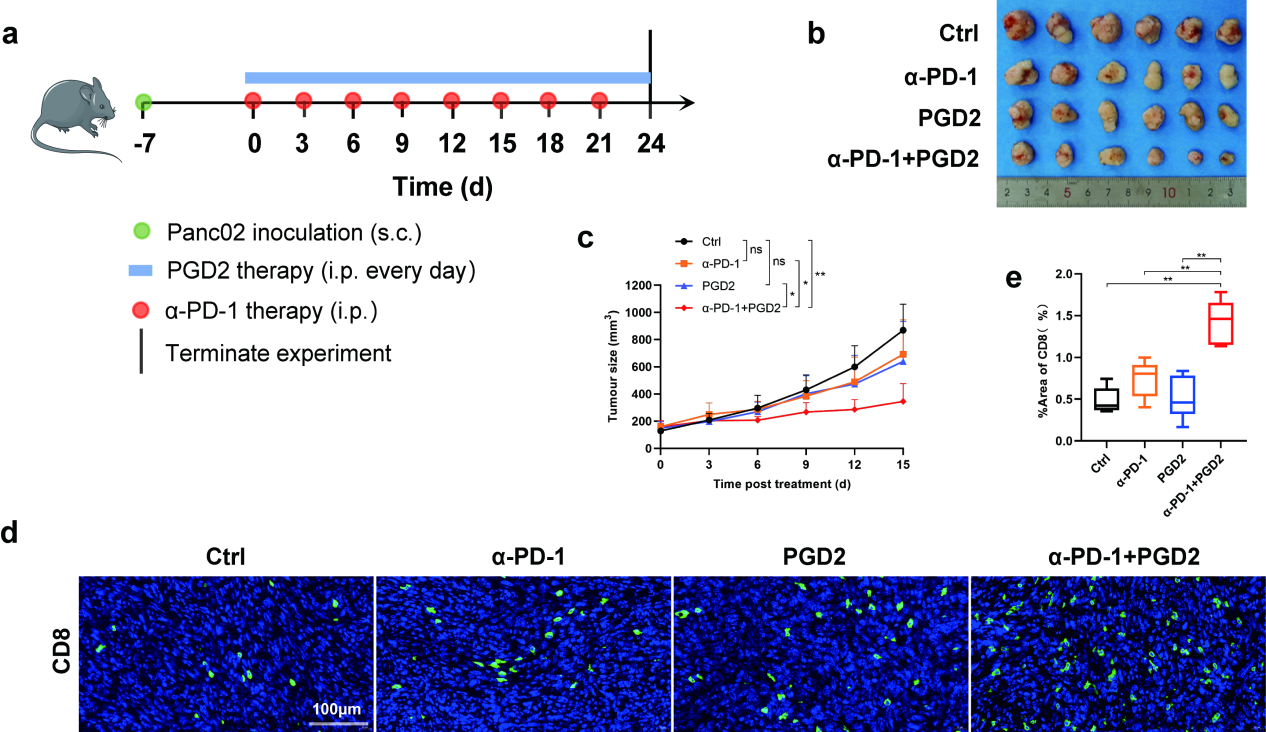


**Supplementary Fig. S5 PGD2 amplified anti-tumor effect of** α-**PD-1 in Panc02 mouse models.** (a) Strategy for the construction of Panc02 mouse model and administration. (b) Tumor tissues of mice in different treatment groups. (c) Tumor growth curves of mice in different treatment groups. (d) Representative images of CD8^+^ T cell in different treatment groups of mice at 400× magnification. (e) Quantitative analysis of the positive area per field by ImageJ software (NIH) using ImmunoRatio plugin.


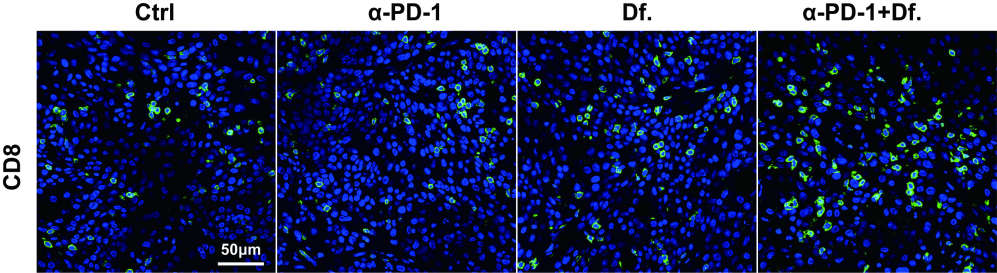


**Supplementary Fig. S6 Representative images of CD8^+^ T cell in different treatment groups of mice at 200× magnification.**
