# Supplementary material for: Resveratrol amplifies the anti-tumor effect of α-PD-1 by altering the intestinal microbiome and PGD2 content
Source: Gut Microbes. 2024 Dec 30;17(1):2447821. doi: 10.1080/19490976.2024.2447821 (PMC12931716; doi:10.1080/19490976.2024.2447821)
Supplement: Supplemental Material [file KGMI_A_2447821_SM3318.zip › Supplementary.docx]

**Supplementary**

**Time (d)**

**0**

**7**

**14**

**21**

**28**

PBMC inoculation (i.v.)

Bxpc3 inoculation (s.c.)

Analyze hCD45^+^ cell in PB


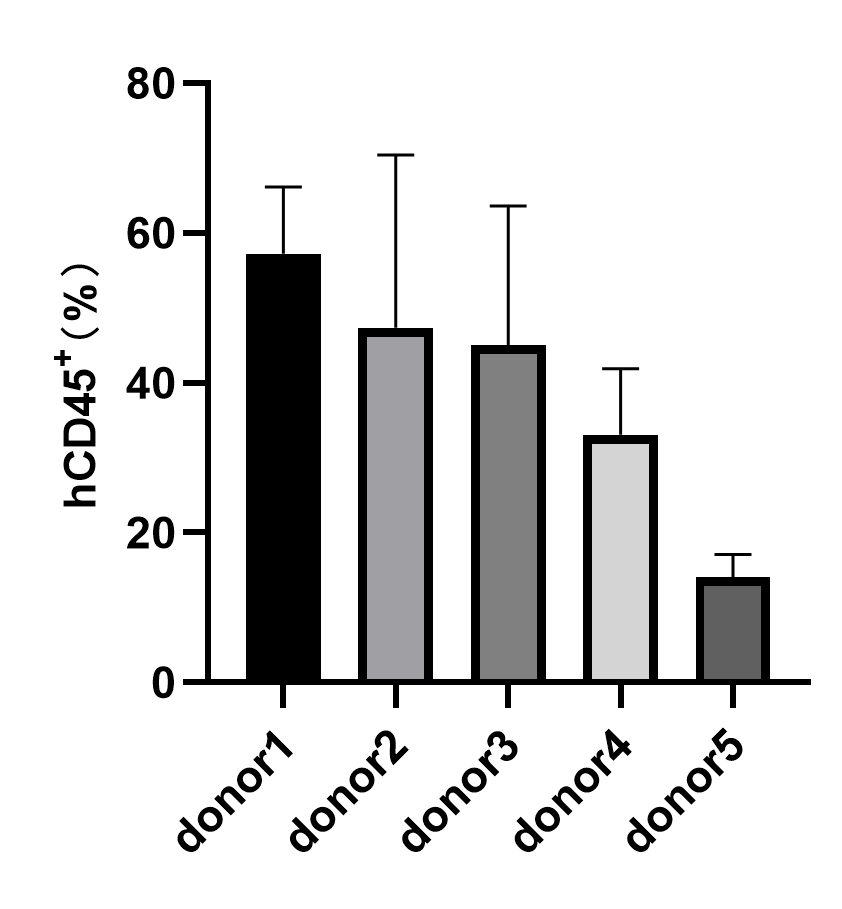

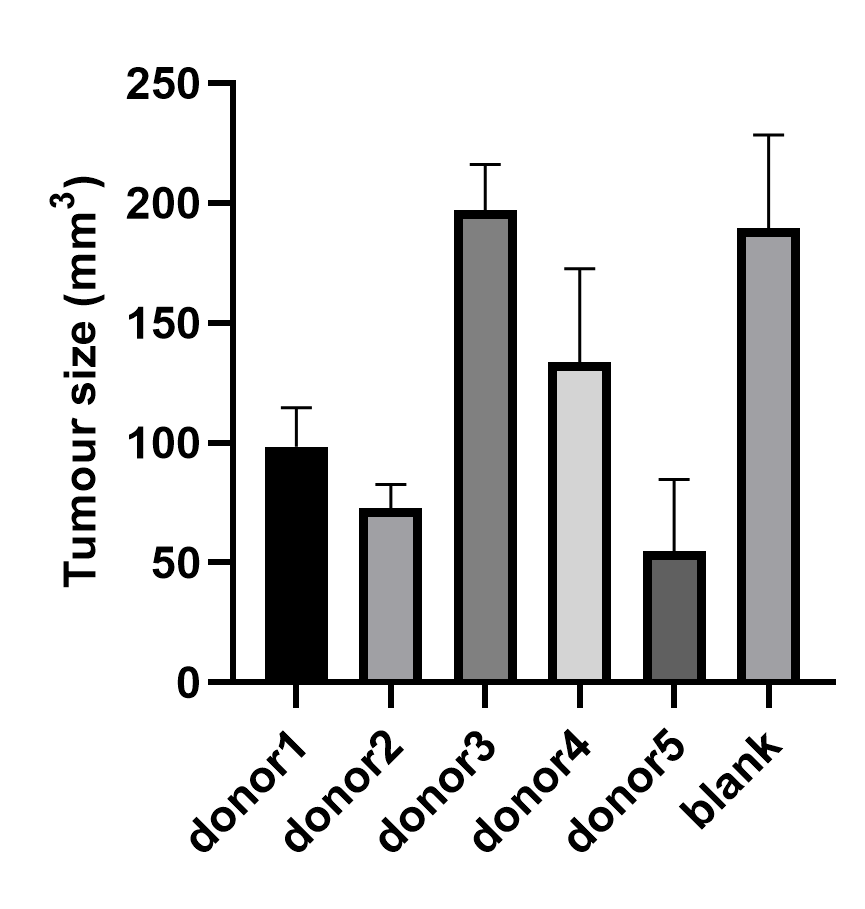


**Supplementary Fig. S1 Identified whether the HLA of PBMC from different donors matched with the pancreatic cancer cell line Bxpc3.** Reconstituted mice by resuscitating frozen PBMCs from different donors to reconstructe human immune system, further transplanted tumor cells, and then evaluated the level of reconstitution and tumor growth so as to identified the histocompatibility between tumor cells and PBMCs.

**Supplementary Fig. S2 loop-gate strategy for flow cytometry.** From left to right, circles indicate all PBMCs of mice, human CD45^+^ cells and mouse CD45^+^ cells from the PBMCs, and human CD45^+^ cells from all CD45^+^ cells.


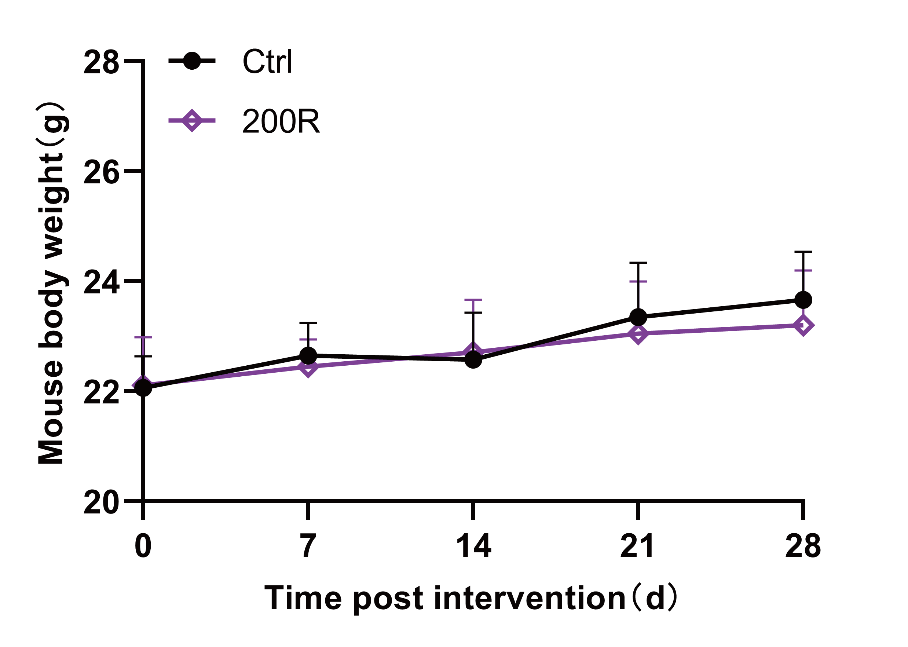


**Supplementary Fig. S3 Curves of mice body weight in the different treatment groups.**


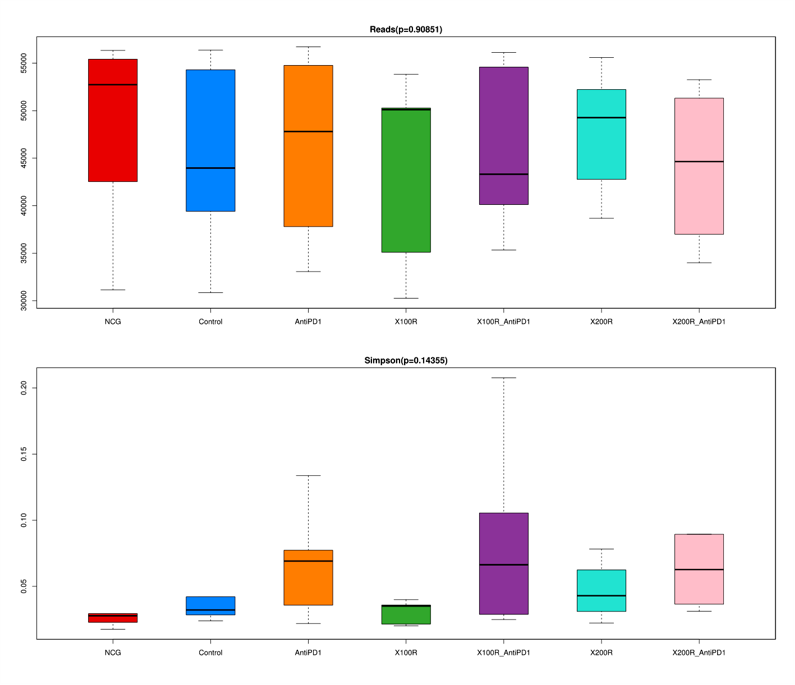


**Supplementary Fig. S4** α**-Diversity of the intestinal microbiota of mice in different treatment groups.**


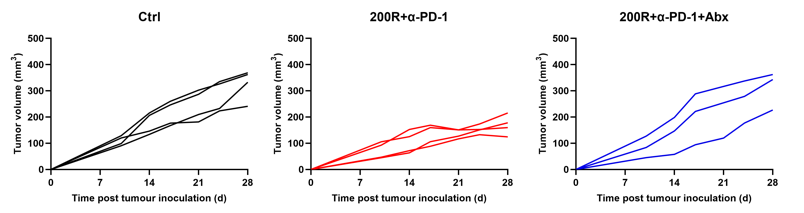


**Supplementary Fig. S5 Tumor growth curves of single mice in different treatment groups.**


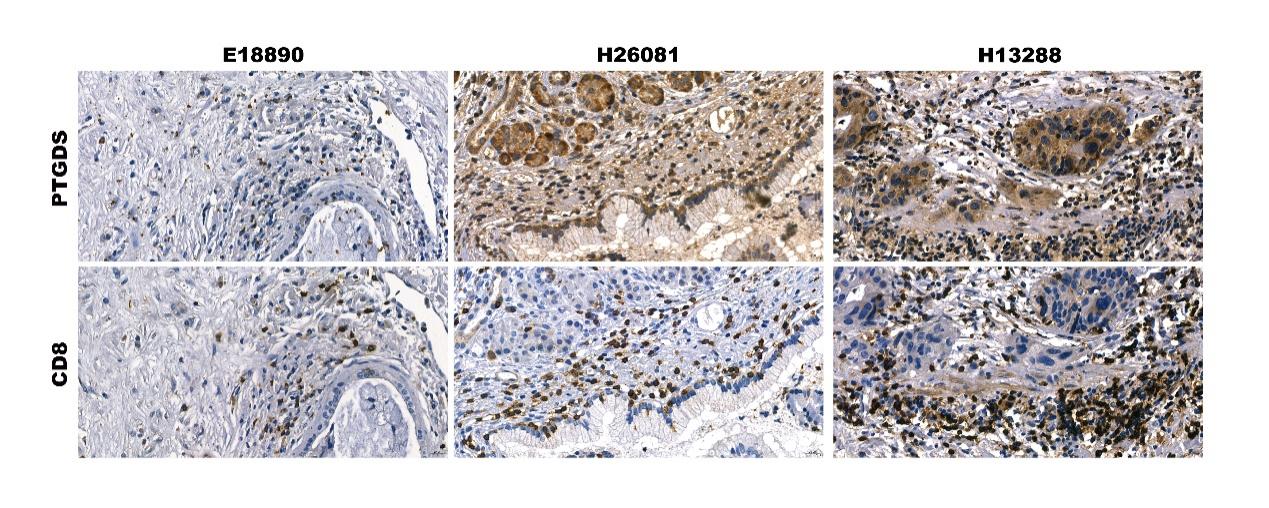


**Supplementary Fig. S6 Representative images of CD8^+^ T and PTGDS in the tumor tissues of different patients with pancreatic cancer at 400× magnification.**


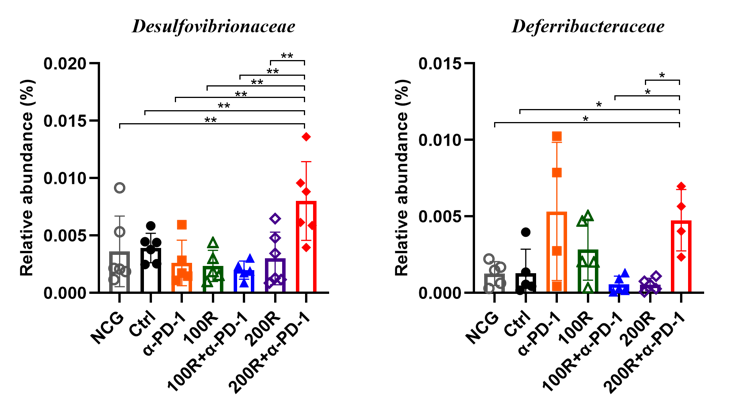


**Supplementary Fig. S7 Relative abundance of Desulfovibrionaceae and Deferribacteraceae in mice in the different treatment groups.**
